# Odontological Society of Pennsylvania

**Published:** 1892-05

**Authors:** 


### ODONTOLOGICAL SOCIETY OF PENNSYLVANIA.

THE usual routine business having been transacted, the President, Dr. Louis Jack, presented the essayist of the evening, Dr. Edward C. Kirk, who gave as the subject of his remarks, "Alveolar Abscess."

(For Dr. Kirk's paper, see page 332.)

*Dr. Darby.*—The case related by Dr. Kirk is exceedingly interesting, and reminds me of one which came under my observation some years ago, and which, I regret to say, terminated fatally. It was that of a gentleman who was much run down in health and in an anæmic condition. He applied to have a broken-down central incisor cut off and an artificial crown grafted upon the root. I found, upon examination, that the pulp was living, and made the usual arsenical application to devitalize it. He called a day or two later, when the pulp was easily and entirely removed, and the root prepared for the crown. An antiseptic dressing was made to the root, and the following day the crown was set. Twenty-four hours later he complained of some soreness in the tooth, which I supposed would readily yield to the ordinary treatment. I saw no more of the gentleman until the following evening, when I was summoned by the family physician to his bedside. I found the patient unconscious, and a malignant alveolar abscess over the root of the tooth in question. I at once lanced and liberated the pus, which was abnormal in color and mingled with dark-colored blood. The patient was unquestionably suffering from pyohæmia. I learned the following day that he was no better, and the day following he died. His physician assured me that the abscess had been the cause of his death, and said that he would probably have died from the prick of a pin had the wound suppurated.

Ten or more years ago a lady called to have the roots of an inferior first molar removed. There was at the time an acute abscess at the apex of one of these roots. After removing one, she concluded that she would defer the extraction of the other. Ten days or two weeks later, I was asked to see her at her house. I found that she had continued to suffer pain, and that the abscess had not been arrested, but the pus had burrowed through the tissues of the neck and had seemed to be drawn by the poultices which she had used constantly lower and lower, until an opening had been made at a point opposite the arm-pit and nipple. It was with the greatest difficulty that she could open her mouth a quarter of an inch. I succeeded in prying her jaws apart, and removed the other root of the offending molar. After a few days' treatment the discharge ceased and the patient recovered. She was a strong, healthy woman at the beginning, but was much reduced in flesh and strength after this prolonged experience. Had she been anæmic, like the first case mentioned, the termination might have been the same.

These are some of the terminations which are liable to occur from apparently simple cases of alveolar abscess.

*Dr. Truman.*—This subject is not one that can well be discussed, as it is simply a relation of facts. It is not a slight matter to have a tooth with alveolar abscess. I remember a case that came into my hands a number of years ago. The patient was taken with acute pericemental inflammation. Having been called out of town for some weeks, his physician attended him. The inflammation became very severe and extensive. What its character was I never learned, but death occurred before I returned. These cases are very unpleasant, as the dentist usually receives all the blame.

I could not quite understand Dr. Kirk's reason for performing an operation externally in the case described. I could not comprehend the necessity for it. If the mouth could be opened, why treat the case externally with poultices? Why not enter the pus-pocket by the mouth? It seems to me that that would have been the proper way. If I had had the treatment of that case, I should have applied cold applications externally, and ice to the jaw, and hot applications in the mouth, and performed the surgical operation there if it should have been required.

Many of these cases come to us after extraction. The relations of the periosteum and pericementum are so intimate that if one be affected the other is necessarily more or less disturbed, and the inflammation that leads to the formation of pus must, in many cases, be continuous. If the socket is closed with coagulated blood and not injected with antiseptics, I cannot understand how this trouble is to be prevented. It seems to me that to avoid these pathological conditions, the greatest care is required in the use of antiseptics after the extraction of teeth.

I criticise Dr. Kirk in the use of the words "laudable pus." There is no such thing as laudable pus; pus is never laudable. I also object to the idea that pus has a definite period for development. Diapedesis of the leucocytes begins very shortly after inflammation is aroused, and will continue to be a factor in the production of pus until normal conditions are established. The part taken in this process by micro-organisms is not now under consideration.

*Dr. J. D. Thomas.*—There are one or two points in Dr. Kirk's statement of the case and in the discussion which impress me quite forcibly. You probably noticed in Dr. Kirk's case that the patient was evidently in a run-down, nervous condition; that his systemic tone was considerably reduced below normal. He did not present an anæmic appearance, yet constitutional conditions were such that violent inflammation or septic infection was bound to follow any exciting cause.

In his case it happened to be a tooth, but I believe the result would have been the same had he received the scratch of a pin upon any part of the body, or a bruise or injury of any other form.

In Dr. Darby's case the patient was in poor health and extremely anæmic. With such conditions there seems to be no limit to which septic infection may follow, either in the retention or removal of a diseased tooth. A member of my own family, while in a poor state of health, was attacked with pulpitis in the right superior second bicuspid, which was treated without success. As a result she lost every tooth back of the canine on that side, accompanied by a perforated antrum.

Fortunately, these extreme cases are very rare, particularly such as the case reported by Dr. Kirk and the one referred to by Dr. Darby; but it is no uncommon thing for inflammation to extend to the surrounding bone and soft tissues, even after the removal of the diseased tooth, resulting in the loss of one or two, or perhaps three, adjoining sound teeth, together with considerable process and bone.

In treating, I have had the best results by encouraging the formation of pus in the inflamed part with hot fomentations until relief from pain is obtained, after which, clearing the sockets of putrescent matter by using antiseptics and astringent lotions, insisting upon systemic treatment in the mean time by their family physician.

*Dr. Jefferies.*—I do not know that I have anything to offer. I have never had a case similar to Dr. Kirk's; but a good many years ago I remember one that, I think, was spoken of before at a meeting of this Society. Perhaps the circumstances attending it will bear repetition at this time; although it was not, strictly speaking, a case of abscess, it resulted in the death of the patient. It came from the treatment of an exposed nerve, and filling a root with gold, preparatory to putting in a crown-filling. The patient lived in Wilmington. The dentist extracted the pulp and filled the root, leaving the crown for a future operation, and sent the patient home; and within twenty-four hours intense pain followed in and about the tooth. It grew so bad towards the evening of the next day that he came to me and implored me to remove it, which, under the circumstances, I did, as there was no possible chance of getting the root-filling out to treat it. It was the next or the second day after that his family physician sent for me to go to his house and lance the gum, thinking there was inflammation, of which I saw no sign. Nevertheless, under his orders the gum was freely lanced. I heard no more of the case until I heard of the man's death. The physician was not of an investigating turn of mind, and I do not know exactly the immediate cause of death. It was evidently in some way connected with the treatment of that tooth. The physician's certificate, I believe, was that death occurred from congestion of the brain.

*Dr. Faught.*—From my early practice I made it a rule not to let my desire to save teeth make me do what might result in a possible fatal termination, where the patient gave evident signs of run-down health, and in the treatment of cases in the lower jaw particularly.

I agree with Dr. Thomas in what he said as to the use of antiseptics. The treatment seems to be simply a treatment with hot water. After extraction in these cases I always advise the use of hot water within the mouth, to bring about the constant stimulation and freest evacuation in the socket. I also long ago adopted the practice of never allowing a tooth to be extracted without having the patient report to me within the next few days, until I am sure all possible tendency to a dangerous result disappears.

I condemn the insertion of any pledgets of cotton in the sockets immediately after the extraction of teeth. The socket should be left freely open, and not have in it anything which will accumulate blood and interfere with the freest flow of pus. I have in many instances been obliged to remove pledgets of cotton put in by others, and to the relief of the patient. I believe the recurrent trouble in almost every instance was due to cotton being packed in the cavity and interfering with the free evacuation of pus.

*Dr. Rehfuss.*—While the discussion has brought out many cases and methods of treatment of various practitioners, still I do not believe that it has shaped itself towards the point that Dr. Kirk most desires, and for which he has brought the subject before us; and that is, what is the duty of the dentist in these cases of alveolar abscess, where we have symptoms of possibly fatal results? All practitioners have cases where there is considerable difficulty in curing them; where possibly an abscess has assumed a chronic form. What the methods of treating are I am not taking into consideration. I cannot at present recall any of my own cases of this kind, but I can remember one of a physician. There was a patient of his who had trouble with a tooth, and in which an alveolar abscess caused septicæmia, and finally death. Now, the physician in this case should have called in the dentist. Dr. Kirk's subject has brought up this question: the duty of the dentist, and the duty of the physician in exceptional cases of this kind. The dentist, in treating a case of alveolar abscess, should follow out his methods in general, and when these constitutional symptoms become pronounced; when he can see or judge that septicæmia or some other condition unknown to him has set in, it is then unquestionably his duty to call some one in consultation who is familiar with diseases of this character. I think that is one point that Dr. Kirk has wished to bring out.

*Dr. Head.*—There has been a great deal said favoring the use of antiseptics after extraction. Whatever may be their value in such cases, I do not think it has been proven. The saliva is a great carrier of germs. No matter how often washes are applied to the socket of the tooth, the diluting saliva will follow, defeating all attempts at antisepsis. In fact, the experiments of Dr. Miller leave the question of their utility in decided doubt.

*Dr. Truman.*—I would like to ask Dr. Head what he would do in gingivitis. The ordinary saliva is constantly running in the mouth, and if he do not use an antiseptic there, I cannot see how he will treat it. There are various preparations that will destroy micro-organisms, and there are others which inhibit their growth. Take, for instance, hydro-naphthol or creolin. If proper solutions of either of these be used continuously, micro-organisms cannot develop in injurious numbers. Clinical experience has demonstrated this over and over again.

In answer to Dr. Thomas, I wish to say, I do not approve of cotton in the socket. I believe in cleaning it out and then treating it antiseptically. I am fully satisfied that all inflammations of the mouth should be treated in this way. I treated a case of necrosis involving the entire right superior maxilla for five months, and it originated from the extraction of a wisdom-tooth.

I can endorse Dr. Thomas's assertion that danger exists of alveolar abscess extending to another tooth. I had an illustration of this that occurred in the clinic of the University this winter. It was that of a young girl, very anæmic, in which the teeth were separated by rubber. The result was inflammation and alveolar abscess in the lateral tooth; it extended to the canine, and, when I saw it, necrosis had taken place. To what extent this went I am not informed, as the patient passed into other hands.

*Dr. Faught.*—There is one sentiment in my mind that I would like to express. It is one in which I have secured statistics with a view to preparing a paper.

A person applies to a dentist with a cavity to be filled; the patient thinks of nothing but a plug to go into the cavity, and all surrounding conditions are entirely lost sight of. It simply reduces itself to a mechanical operation. We get the same idea too frequently, I think, in regard to alveolar abscess. I am speaking of the acute form.

The sentiment is simply this: That to my mind alveolar abscess belongs to that class of surgical operations best treated at the home of the patient, and that the dentist should become a visitant until acute symptoms have abated, and that systemic conditions should never be omitted in its treatment when purely local treatment is not meeting the requirements of the case. We too often look only to local treatment. I think we ought to relegate this acute form to the room and bed, where the case can be managed by visitation.

*Dr. Thomas.*—Dr. Kirk calls attention to the efficacy of phénol sodique. I agree with him in its applicability to cases after extraction. It is one of the best antiseptic remedies we have; but in cases of acute periostitis, where the pain continues for some hours after extraction, its use is contra-indicated, and hot fomentations are the more desirable. After the inflammation has reached its height and is at a stand-still, then by washing the cavity and applying a loose pledget of cotton saturated with phénol, you have, to my mind, the best means of rapid recovery extant.

*Dr. Jack.*—I have not had a case similar to the one Dr. Kirk has referred to. I would only express caution when serious peridental inflammation occurs in the lower maxilla, and would call especial attention to the danger in cases of purulent inflammation connected with the inferior wisdom-tooth.

It may be known to some of you that a few years since there happened a case, in the upper part of Philadelphia, of the death of a patient under the care of a physician, where the cause of death was assumed to be tonsillitis, but it was found, as the result of the autopsy, that the death was due to the occurrence of abscess of the inferior wisdom-tooth, the result of which was the infiltration of tissues at the base of the tongue. The pus had burrowed along the course of the muscles, and had very freely infiltrated the tissues and the hyoid bone with pus. The patient died of pyæmia.

In my experience a case occurred of a prominent clergyman who came to me with inflammation of a lower wisdom-tooth, and it had a very unpleasant appearance and was already threatening, as I thought, to burrow along the mylo-hyoid. I said to him, "This tooth should be removed;" but he was a man of determined disposition, and said he would not have it out. In the course of a few days his physician sent for me and wished me to visit the patient. When I arrived at the patient's house I found extreme evidence that there was infiltration of pus. I said that the tooth should be removed at once. The response was that the condition of the patient was such that it would be dangerous to health and life to attempt it under the circumstances. My response was that if the tooth was not quickly extracted, the most disastrous consequences might ensue. The tooth was removed, and I may state to you that the patient was confined to his bed for some six weeks. The physician treated him for a conditon similar to pyæmia.

I only cite this case, not that it bears any direct resemblance to those given, but to state my view that in cases of severe inflammation of the peridental membrane of the lower wisdom-tooth, it should be extracted at the earliest possible moment after the abscess condition becomes indicated.

*Dr. McQuillan.*—I would like to ask this question: How long are we warranted in treating a devitalized tooth, particularly those that have blind abscesses? We have all had experience in filling teeth, and dismissing patients and telling them they can go for six months, and having them come back the next day; and the question has come to me, How long are we warranted in treating these teeth when it is impossible to get a fistulous opening, and after the tooth is filled is it necessary to remove the filling to continue the treatment? It seems to me, particularly in the case of the lower molars, that at times I am running great risk in continuing very long treatment.

*Dr. Kirk.*—I would like, if I may be allowed, to give a word of explanation at this point that I hope will serve to direct the trend of the discussion on this subject. I omitted a full description of what was done when the patient returned after the extraction of the tooth. The omission was accidental. I washed out the socket with water; I did sterilize it as fully as I knew how, but not with corrosive sublimate; I used carbolic acid in full strength. I did endeavor to evacuate the pus through the bottom socket by passing a sharp-pointed instrument down as far as I dared. I stated in the description, I think, that the jaw was unusually deep. There was a distance of an inch or an inch and a quarter between the upper border of the alveolar ridge and the free lower border of the body of the maxilla. This accumulation of material, whether pus or not, was situated on a line immediately below the socket of the sixth-year molar and upon the border of the bone. If I had waited to carry out the treatment such as Dr. Truman suggests, and which I would use in a majority of cases, I believe the patient would have died before an evacuation of the accumulated pus could be brought about within the mouth.

In speaking of laudable pus, I should have qualified it by saying, so-called laudable pus. I agree with Dr. Truman, that there is in a certain sense no such thing as laudable pus. I used the term in its popular sense, in order to make clear my description of the kind of pus referred to.

I would be glad if Dr. Truman had explained a little more fully just what constitutes pus. It is yet an open question. I have not a full and definite idea of what constitutes pus; but the diapedesis of leucocytes has been shown to be but one factor in its formation. Some source of irritation is needed to account for the diapedesis, and I think almost all pathologists agree that it is generally the result of some infection.

The reason I did not operate from the inside was because it was clear to my mind, from a careful examination, that I could not establish a free evacuation of pus by that channel, and it was also evident that the condition with which I had to deal was one of those pathogenic infective inflammations which was likely to produce pyæmia. I was actuated by my desire to prevent the man from dying, and by the belief that, in order to avoid a possible fatal result, the inflammatory products should be evacuated as soon as possible.

The incisions made on the outside of the face in this case have healed up almost without a scar. They were made with a very sharp knife, and such an incision always makes but a slight scar. I believe I materially shortened the course of the disease, if I did not absolutely save the patient's life, by the operation as described.

I brought a report of this case before the Society for two reasons. I think we are inclined to group under the general term, alveolar abscess, a number of diseases which are related only by reason of their position, and which have the factor in common with many other acute inflammations, the resulting discharge of pus. But, I judge, from the investigations of Dr. Miller and others who have written upon the subject, that it is time that we recognized that there is a difference in these diseases. Alveolar abscess is a general term. I take it for granted that we have all of us read enough on this subject to warrant us in concluding that the character of inflammatory action is determined largely by the nature of the germs which produce it. One of the diseases which I have outlined to you this evening is what is known, when it occurs apart from the jaws, as phlegmonous inflammation. It is an inflammation which is closely related to erysipelas, and is a quite different disorder from ordinary alveolar abscess, and is frequently fatal, as shown by the number of cases that are reported by Dr. Miller in the reprint from the *Dental Cosmos* which I have distributed. I have had these reprints made for this purpose simply because I was interested in getting an expression from the members of this Society on this very important subject.

I firmly believe that if I had not carried out the treatment in this case upon general surgical principles, I would have lost my patient. It was not alone because the patient was run down that this particular form of abscess occurred, but largely because of the specific nature of the infection.

As to cotton, I would like to say one word in favor of it. Of course, plain cotton may and does, inside of a few hours, become an infectious mass. My use of it was simply as a vehicle for retaining antiseptics in contact with the wound. If the socket is left open, the next time you wash it out you will have a collection of food *débris* in a state of decomposition, which, to my mind, is much more offensive than that of cotton-pledgets properly saturated with some antiseptic. With regard to suitable antiseptics, we have phénol sodique. Without attempting to give a scientific explanation, I know, from practical experience, that nothing I have used has acted so kindly as an application to the sockets, after the extraction of teeth, as this agent. I do not approve of leaving cotton-dressings in the sockets for an indefinite time; therefore, the patient is given directions to remove it. If not able to do so, he is directed to come back to me, to have the socket redressed in a similar manner, until healing is well established.

Under such treatment I get better results than where a simple mouth-wash is used. By the means of cotton-pledgets I bring the antiseptic into close contact with the tissues I wish to sterilize.

